# Acute left main coronary artery thrombosis due to cocaine use

**DOI:** 10.1186/1749-8090-5-65

**Published:** 2010-08-19

**Authors:** Efstratios Apostolakis, Grigorios Tsigkas, Nikolaos G Baikoussis, Ioanna Koniari, Dimitrios Alexopoulos

**Affiliations:** 1 Cardiothoracic surgery Department, Patras University School of Medicine, University Hospital, Patras Greece; 2 Cardiology Department, Patras University School of Medicine, University Hospital, Patras Greece

## Abstract

It is common knowledge that cocaine has been linked to the development of various acute and chronic cardiovascular complications including acute coronary syndromes. We present a young, male patient, drug abuser who underwent CABG due to anterolateral myocardial infarction. Our presentation is one of the very rare cases reported in literature regarding acute thrombosis of left main coronary artery related to cocaine use, in a patient with normal coronary arteries, successfully operated. Drug-abusers seem to have increased mortality and morbidity after surgery and high possibility for stent thrombosis after percoutaneous coronary interventions, because of their usually terrible medical compliance and coexistent several problems of general health. There are no specific guidelines about treatment of thrombus formation in coronary arteries, as a consequence of cocaine use. So, any decision making concerning the final treatment of these patient is a unique and individualized approach. We strongly recommend that all these patients should be treated surgically, especially patients with thrombus into the left main artery.

## Introduction

The first relationship between myocardial ischemia or acute coronary syndrome and cocaine use, was reported by Coleman D, et al, in 1982 [1]. Many studies have investigated and documented the mentioned relationship concerning its pathophysiology and management. The risk of acute myocardial infarction (AMI) is increased 24-fold during the first hour after cocaine use in patients with normal coronary arteries (CA) [2]. According to several studies, most cocaine-abusers with myocardial related complications are young, smokers, nonwhite and male, without other risk factors for atherosclerosis [3]. Therefore, when young patients with no or few risk factors for atherosclerosis, are present with AMI, they should be questioned about cocaine use, and urine plus blood samples should be analyzed for cocaine and its metabolites. Cocaine induces vasoconstriction of epicardial CA in patients with or without coronary artery disease (CAD), combined with an enhanced platelet activation and aggregation, leading in thrombus formation [4]. Of note, in most of the reported cases the affected CA is a distal coronary branch and not the left main coronary artery (LM). We describe herein a LM thrombosis in a young cocaine-abuser, who underwent emergency coronary artery bypass grafting (CABG) for persistent post-infarction angina and acute heart failure.

## Case presentation

**Figure 1:**
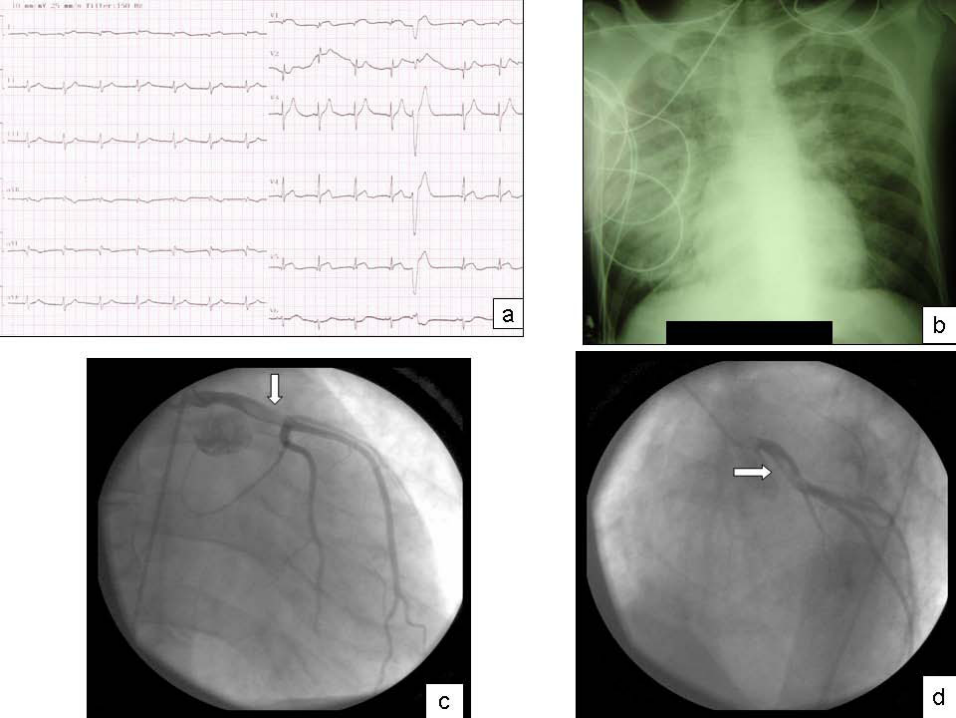
**An electrocardiographic, x-ray and angiographic picture of our patient are present showing the severity of the condition. A**. Electrocardiogram shows QR complexes in leads I, aVl, V1 and V2 due to anteroseptal and lateral myocardial infarction caused by a proximal Left Anterior Descending artery occlusion, involving septal and marginal branches. Moreover, one could recognize mild ST-T segment elevation in leads I, aVl, V4, V5. **B**. Common signs for acute heart failure after a large acute myocardial infarction can be seen on chest x-ray, as alveolar and interstitial edema and prominent upper lobe vessels. **C, D**. Cocaine abuse and thrombosis of Left coronary system. Right anterior oblique (RAO) and left anterior oblique (LAO) projection of Coronary angiogram revealing thrombus (arrows) formation in Left main artery.

A 28-year-old man smoker and substance abuser (cocaine and circumstantial intravenous heroin) for the last 8 years, without known familiar history for CAD, presented to a local hospital with an antero-lateral ST segment elevation myocardial infarction (Figure 1A), associated with a low cardiac output syndrome. Heart ultrasound revealed severe hypokinesia of anterolatelar wall with ejection fraction of 30%. The patient was given immediately thrombolysis with tenecteplase (Metalyse) and his symptoms and ECG changes almost resolved. Few hours later he was transferred to our hospital for urgent catheterization due to recurrent ischemia and hemodynamic instability. At his admission the patient suffered from AMI complicated with cardiogenic shock and symptoms of congestive heart failure. Chest x-ray examination showed mild cardiomegaly, diffuse pulmonary congestion and perihilar infiltrates with the classic butterfly pattern, and Kerley B lines (Figure 1B). He was supported medically by combination of dobutamine, dopamine and furosemide. Coronary angiography revealed a huge thrombus formation of LM and suspicion of mobile parts to the rest of the left coronary system (Figures 1C, D). He stabilized hemodynamically using an intraaortic balloon pump (IABP), but due to a lot of episodes of angina and no clear-cut guidelines describing the best management approach for patients under this condition we decided to operate him under an estimated risk of 30% according to logistic EURO-score. Urgent CABG by using cardiopulmonary bypass at normothermia (37°C) with continuous warm blood cardioplegia was done. An organized thrombus was found in the opened left anterior descending artery and removed. Finally, the vessel was bypassed by the left internal thoracic artery. In addition, the first obtuse marginal branch was bypassed by a free left radial graft. After a successful bypass weaning, he was transferred in intensive care unit and extubated 12 hours later. The IABP was removed 24 hours later. Inotropes infusion was interrupted at 36 hours and he discharged from the hospital on the 8^th ^postoperative day in a good condition.

## Discussion

Three factors are implicated in the pathogenesis of cocaine-related myocardial ischemia-infarction: the increased myocardial oxygen demand, the marked vasoconstriction of CA, and the exaggerated platelet aggregation [3,4]. Cocaine is a powerful sympathomimetic and dramatically increases oxygen demand by blocking the reuptake of norepinephrine and dopamine at the presynaptic adrenergic terminals. Also, induces significant increase of myocardial oxygen demand due to increased heart rate, systemic arterial pressure and left ventricular contractility. The chronotropic effects of cocaine are intensified in the setting of alcohol use. In addition, cocaine produces a significant coronary vasoconstriction either in normal CA or -more marked- in diseased one [5]. Factors that are implicated to this coronary vasoconstriction are the increased endothelial production of endothelin and the decreased production of nitric oxide [6]. There are a lot of reported cardiovascular complications of cocaine users but the incidence of most severe of them is relatively uncommon. According to Hollander J, et al [7], ventricular arrhythmias occurred in 4 - 17%, congestive heart failure in 5 - 7%, and death in <2%. Therefore, the incidence of cocaine abusers who will need an emergency interventional or surgical reperfusion is low. Interventions should be directed either to treat ischemic complications due to thrombosis [1-3], dissection [8], or acute/subacute thrombosis of an implanted coronary stent [9]. Vasoconstrictive and atherosclerotic effects of cocaine, combined with the well documented platelet-activating effect, significantly increase the post-PCI risk of stent thrombosis (ST), in the early and late post implantation phase. Karlsson G et al [9], reported that among the actively using cocaine patients who underwent PCI, 33% of them presented with ST 51 ± 40 days after the intervention, with an incidence of ST almost 10-fold higher in cocaine users. Similar results reported in a retrospective study by Singh S, et al [10]. Of their patients with active cocaine use 5% suffered SF with a mean period from stent implantation 28.5 ± 14 days.

Our presentation is one of the rare cases reported in literature referring in AMI due to thrombosis of LM related to cocaine use, in a young patient with normal CA, successfully operated. Drug-abusers seem to have increased mortality and morbidity because of their usually coexistent several problems of general health. Furthermore, a majority of patients that suffer ST continue cocaine and are noncompliant with medical therapy. Improved handling techniques and postoperative long term dual antiplatelet therapy additionally to special consultation services could reduce the incidence of this terrible event. The lack of specific guidelines about treatment of thrombus formation in CA, especially in LM, a catastrophic consequence of cocaine use, leads to a unique and individualized approach to these patients. The small number of reported cases prevents the development of an evidence-based management, but due to high probability of post PCI ST, as mentioned before, we strongly recommend that all these patients should be treated surgically.

## Competing interests

The authors declare that they have no competing interests.

## Authors' contributions

All authors: 1. have made substantial contributions to conception and design, or acquisition of data, or analysis and interpretation of data; 2. have been involved in drafting the manuscript or revisiting it critically for important intellectual content; 3. have given final approval of the version to be published.

## Consent

Written informed consent was obtained from the patient for publication of this case report and accompanying images. A copy of the written consent is available for review by the Editor-in-Chief of this journal.
